# Clinical, histological and molecular predictors of metastatic melanoma responses to anti-PD-1 immunotherapy

**DOI:** 10.1038/s41416-018-0168-9

**Published:** 2018-07-05

**Authors:** Frantz Dupuis, Laurence Lamant, Emilie Gerard, Nouritza Torossian, Leonor Chaltiel, Thomas Filleron, Marie Beylot-Barry, Caroline Dutriaux, Sorilla Prey, Audrey Gros, Marie-Laure Jullie, Nicolas Meyer, Béatrice Vergier

**Affiliations:** 1 Department of Pathology, Hôpital Haut-Lévêque (CHU de Bordeaux), 33604 Pessac, France; 2 Department of Pathology, Oncopole de Toulouse, 31100 Toulouse, France; 3 Université Paul-Sabatier, 31400 Toulouse, France; 4 Department of Dermatology, Hôpital Saint-André (CHU de Bordeaux), 33000 Bordeaux, France; 5 Department of Dermatology, Paul-Sabatier–Toulouse III University (CHU de Toulouse), 31059 Toulouse, France; 6 Department of Biostatistics, Oncopole de Toulouse, 31100 Toulouse, France; 7 INSERM U1053 Team 1 (université de Bordeaux), 33076 Bordeaux, France; 8 Department of Tumour Biology and Tumour Bank, Hôpital Haut-Lévêque (CHU de Bordeaux), 33604 Pessac, France

## Abstract

**Background:**

Prescribing anti-programmed death-1 (PD-1) immunotherapy for advanced melanoma is currently not restricted by any biomarker assessment. Determination of programmed death-ligand-1 (PD-L1)-expression status is technically challenging and is not mandatory, because negative tumours also achieve therapeutic responses. However, reproducible biomarkers predictive of a response to anti-PD-1 therapy could contribute to improving therapeutic decision-making.

**Methods:**

This retrospective study on 70 metastatic melanoma patients was undertaken to evaluate the relationships between clinical, histological, immunohistochemical and/or molecular criteria, and the 6-month objective response rate.

**Results:**

Better objective response rates were associated with metachronous metastases (*P* = 0.04), PD-L1 tumour- and/or immune-cell status (*P* = 0.01), CD163+ histiocytes at advancing edges (*P* = 0.009) of primary melanomas and *NRAS* mutation (*P* = 0.019). Moreover, CD163+ histiocytes at advancing edges (*P* = 0.04) were associated with longer progression-free survival (PFS), and metachronous metastases with longer overall survival (*P* = 0.02) and PFS (*P* = 0.049).

**Conclusions:**

Combining these reproducible biomarkers could help improve therapeutic decision-making for patients with progressive disease.

## Introduction

Melanomas are highly immunogenic cancers,^1^ whose prognoses have been dramatically improved by immunotherapy. Nivolumab or pembrolizumab anti-programmed death-1 (PD-1) monoclonal antibodies have been reported to significantly increase advanced melanoma patients’ response rates, progression-free survival (PFS) and overall survival (OS), compared to anti-cytotoxic T-lymphocyte antigen-4 antibodies and/or chemotherapy.^2–5^ Overall response rates (ORRs) in pivotal clinical studies on nivolumab or pembrolizumab, respectively, were 28%^6^ or 38%.^7^ Anti-PD-1 is currently indicated as first-line treatment for *BRAF*– (v-Raf murine sarcoma viral oncogene homolog B) metastatic melanomas or sometimes *BRAF*+ ones with few metastases or slow evolution.

Tumour-cell programmed death-ligand-1 (PD-L1) expression has been associated with better outcomes under anti-PD-1 immunotherapy: a recent meta-analysis highlighted better odds ratios of ORRs for the 1%- and 5%-positive tumour-cell thresholds (respectively, 2.81, *P* = 0.0002; and 2.22, *P* < 0.00001).^8^ Moreover, because all studies reported significant response rates of PD-L1– melanomas,^9^ PD-L1–status determination is not mandatory for the prescription of these agents to melanoma patients, unlike lung adenocarcinomas.^10^

Biological and technical challenges in immunohistochemically evaluating PD-L1 expression might explain, at least partially, PD-L1 status-therapeutic response discordances,^11^ because tumour and immune cells must be considered, and the latter are frequently PD-L1+.^12^ Moreover, other cells, e.g. CD8+ tumour-infiltrating T lymphocytes (TILs)^13^ or CD163+ histiocytes,^14^ that play important roles immune-response regulation, could be involved in immunotherapy efficacy.

We hypothesised that PD-L1-expression status combined with other clinical, histological and/or molecular criteria might be able to better predict responses to anti-PD-1 therapy than PD-L1 status alone. Therefore, we retrospectively analysed characteristics of 70 anti-PD-1-treated, metastatic melanoma patients, to try to identify markers contributive to therapeutic decision-making.

## Materials and methods

This two-centre (Toulouse and Bordeaux), retrospective, biomarker analysis concerned a cohort receiving routine care. Seventy patients with advanced and/or metastatic cutaneous melanoma and available formalin-fixed, paraffin-embedded (FFPE) tumour blocks were identified. All primary and/or metastatic lesion specimens were collected before any anti-PD-1 administration. All patients provided written informed consent for the use of the samples, and the local Ethics Committee approved the study. Clinical and histological parameters and follow-up data were retrieved from medical files. The primary outcome measure was the 6-month ORR, determined radiologically with iRECIST criteria.^15^ We also collected acute (first month) and chronic (thereafter) immune adverse events (IAEs), defined as all AEs, most frequently skin, bowel, liver or endocrine symptoms potentially attributable to immunotherapy.

### Immunohistochemistry

An automated Leica immunohistochemistry instrument labeled 3-*µ*m-thick sections of FFPE blocks with E1L3N (Cell Signaling Technology, Leiden, The Netherlands; diluted 1:100). As stipulated in REMARK criteria, for each case, anti-PD-L1–antibody 22C3-immunolabeling (Dako kit, Glostrup, Denmark) was run in parallel on an automated Dako immunohistochemistry apparatus, for comparison with the more novel E1L3N.

**Fig. 1:**
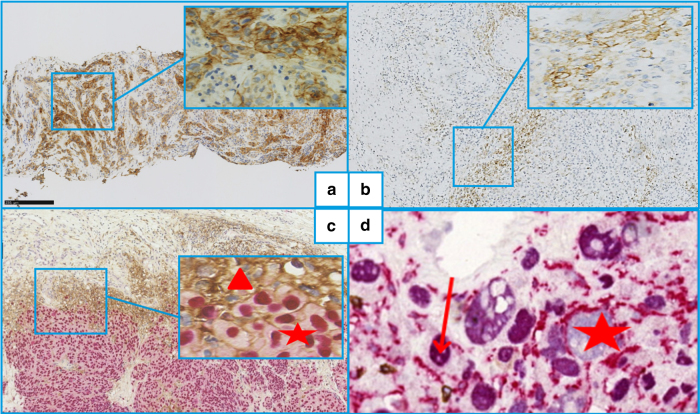
Immunohistochemical labeling of metastatic melanoma primary lesions or metastases. **a** All tumour (melanoma) cells are programmed death–ligand-1 (PD-L1)–positive, which is quite rare (×100, E1L3N clone). **b** A sample with >5% PD-L1–positive tumour area. Note: the disposition of the PDL1+ cells at the tumour edge is very common (×100, E1L3N clone). **c** SOX10 (purple nuclei) and PDL1 (brown membranes) double-labeling. Note: PD-L1+ is expressed both by immune cells (SOX10 negative, red triangle) and tumour melanocytes (SOX10 positive, red star) (×200). **d** SOX10 (purple nuclei, red arrow) and CD163 (brown membranes, red star) doublelabeling. Note: CD163-labeling of histiocyte cytoplasmic extensions surrounding SOX10+ tumour cells could suggest that some PD-L1+ cells thought to be tumoural might rather be PD-L1– tumour cells surrounded by positive histiocyte cytoplasmic extensions (×200)

The percentages of PD-L1+ tumour cells and tumour-area (including tumour and/or immune cells), and PD-L1+ cells and CD163+ (clone 10D6, Leica) histiocytes at advancing edges (immediate tumour periphery) of invasive melanomas, were determined as previously described.^8,16^ Positivity was defined as PD-L1 cell-membrane labeling of >5% of tumour area and cells, and >10% of PD-L1 and CD163+ histiocytes at advancing edges (Fig. 1). The advancing tumour edge was defined as the tumour cell–peritumour-inflammation interface, without specifying width. Those values were evaluated semi-quantitatively, by light microscopy of the whole slide, not only a region, first assessing PD-L1-labeling intensity with four-tier grading ( + to + + + ), but, because intensity of the vast majority of samples did not vary, we chose not to use it. PD-L1 cut-offs were chosen based on the literature findings, and those for CD163 were derived from our pathologists’ preliminary study of 10 characteristic cases.

PD-L1 expressions in each patient’s primary melanoma and metastases were compared. TILs, their intratumoural or peritumoural distribution pattern (brisk, with T cells throughout the lesion or at its outer edge, or non-brisk, not involving the entire lesion or outer edge) and CD8 (clone 144B, Dako) expression were analysed according to the literature.^17,18^ All criteria were independently assessed by three experienced dermatopathologists (FD, BV, LL) blinded to clinical-radiological information. Between-observer scoring mismatches were resolved by case review.

### Statistical analyses

Categorical variables, expressed as numbers, (%) and continuous variables, as median [range], were compared respectively using *χ*^2^ or Fisher’s exact tests and Kruskal–Wallis tests. All survival parameters were measured from the date of the first anti-PD-1 infusion. Kaplan–Meier analyses of PFS and OS used the following first-event definitions, respectively: progression or death, and death. Univariate analyses used the log-rank test, with *P* *≤* 0.05 defining significance. Statistical analyses were computed using STATA and R software. Given the exploratory nature of this study, no adjustments were made for multiple comparisons.

Via recursive partitioning (rpart package in R v4.1–10), classification-and-regression trees created a decision tree indicating how well different variables predict 6-month objective response class (confusion matrix available in supplementary figure 9). The information index was used for the splitting rule to stratify data into subsets of individuals, represented as nodes in the decision tree, with the package’s default option imputing missing values. Positive- (PPV) and negative-predictive values (NPV) were calculated.

The intraclass-correlation coefficient (ICC) assessed interobserver variability of PD-L1-expression scores.

## Results

Seventy patients’ 118 melanoma samples were examined; 48 had primary and metastatic specimens available (Table 1). Metastasis locations were: 26 in skin, 34 in lymph nodes, two in liver, two in lung and six elsewhere. Pembrolizumab was given to 72.9% of the patients and nivolumab to 27.1%. Median follow-up was 19.3 (95% confidence interval (CI): 16.2–21.4) months. The 6-month ORR was 28.6%, with 69.7% (57.42–79.09%) OS, median PFS lasted 5.75 (2.99–10.28) months, and median OS 15.9 (95% CI: 8.7–not reached) months. Neither IAE severity nor frequency was associated with ORR or > 5% PD-L1+ tumour areas. Among the clinical characteristics analysed, only synchronous vs metachronous metastases differed significantly for ORR, OS and PFS (Kaplan–Meier plots available in supplementary figures 3 and 7).

**Table 1:** Univariate analyses of characteristics of the 70 metastatic melanoma patients given anti-PD-1 immunotherapy as a function of outcome measures

Characteristic	Value^a^	6-month ORR (%)	Overall survival (%)		PFS (median, months)
			6 months	12 months	
**Clinical**					
*Gender*		NS			
Male	58.6 (41)	31.7			
Female	41.4 (29)	24.1			
*Age (years)*		NS			
<60	38.6 (27)	37			
>60	61.4 (43)	23.2			
*Lactate dehydrogenase*		NS	NS		NS
<1 Normal	37.5 (18/48)	38.8	76.5	70.6	11.7
>1 Normal	62.5 (30/48)	20	70	56.7	5
*Metastases*					
Chronology		*P*=0.04	*P*=0.002		*P*=0.049
Synchronous	27.1 (19)	10.5	36.8	31.6	3
Metachronous	72.9 (51)	35.3	82	65.14	10.3
Months to onset, median (range)	24 (1.1–219)	NS			
*Previous treatments*		NS	NS		NS
None	25.7 (18)	33.3	77.78	60.6	8.9
1 treatment	41.4 (29)	20.7	68.14	53	5
>1 treatment	32.9 (23)	34.8	65.22	56	5.6
*Anti-PD-1 immunotherapy*					
Treatment line, median (range)	2 (1–8)	NS			
Molecule		NS			
Pembrolizumab	72.9 (51)	27.45			
Nivolumab	27.1 (19)	31.6			
Days of treatment, median (range)	148.5 (7–745)	*P*≤0.0001			
Number of cycles, median (range)	10.5 (1–39)	*P*≤0.0001			
*Immune adverse events*					
Acute	21.4 (15)	NS			
Chronic	50 (31/62)	NS			
*Follow-up (all patients)*					
6-month ORR, % (*n*)	28.6 (20)				
Median follow-up (months)	19.3				
12-month OS (%)	56				
Median OS (months)	15.9				
Median PFS (months)	5.75				
Alive at the end of the follow-up, % (*n*)	50 (35)				
**Morphological and molecular**					
*Primary tumour*					
pT		NS	NS		NS
T1/T2	32.3 (20/62)	20	60	40	3.3
T3/T4	67.7 (42/62)	31	76	63.1	5.7
pN		NS	NS		NS
N0/N1	42.4 (25/59)	36	67	57.5	5.7
N2/N3	57.6 (34/59)	23.5	73.5	61.8	5.6
pM		NS	NS		NS
M0	34.3 (24)	33.3	70.8	51.5	5.7
M1 (a, b or c)	65.7 (46)	26	69.1	58	5.6
TILs		NS			
Yes	75 (36/48)	16.7			
No	25 (12/48)	25			
Brisk TILs		NS			
Yes	66.7 (24/36)	16.7			
No	33.3 (12/36)	29.1			
Brisk TIL pattern		NS			
Intratumoural	45.2 (14/31)	21.4			
Peritumoural	54.8 (17/31)	35.3			
Other features					
Ulceration	44.3 (27/61)	NS			
Median (range) Breslow, mm (*n*=63)	3.3 (0.2–15)	NS			
PD-L1+ tumour area		*P*=0.02	NS		NS
>5%	58.3 (28/48)	35.7	59.6	48.4	3.9
<5%	41.7 (20/48)	5	85	70	8.4
PD-L1+ at advancing edges		NS	NS		NS
>10%	46.8 (22/47)	31.8	57.75	43.3	2.8
<10%	53.2 (25/47)	16	84	72	8.9
PD-L1+ tumour cells		NS	NS		NS
>5%	10.4 (5/48)	40	74.1	40	3
<5%	89.6 (43/48)	21	40	60	5.6
CD163+ cells at advancing edges		*P*=0.009	NS		*P*=0.04
>10%	68.1 (32/47)	34.3	73.3	61.5	8.4
<10%	31.9 (15/47)	0	71.3	53.3	2.8
*Metastasis*					
TILs		NS			
Yes	57.9 (22/38)	31.8			
No	42.1 (16/38)	25			
Brisk TILs		NS			
Yes	63.6 (14/22)	28.6			
No	36.4 (8/22)	37.5			
Brisk TIL pattern		NS			
Intratumoural	58.8 (10/17)	40			
Peritumoural	41.2 (7/17)	14.3			
PD-L1+ tumour area		NS	NS		NS
>5%	61.4 (43)	30.2	62.8	51.1	8.9
<5%	38.6 (27)	25.9	81	63.8	5
PD-L1+ at advancing edges		NS	*P*=0.025		NS
>10%	46.2 (30/65)	20	50	43.3	3
<10%	53.8 (35/65)	34.3	85.4	63	8.4
PD-L1+ tumour cells		NS	NS		NS
>5%	21.4 (15)	46.6	60	46.7	8.7
<5%	78.6 (55)	23.6	72.4	58.7	5.6
CD163+ cells at advancing edges		NS	NS		NS
>10%	53.8 (35/65)	25.7	64.9	49	3.9
<10%	46.2 (30/65)	30	76.7	66.7	8.4
Primary tumour and metastasis agreement					
PD-L1+ tumour area	43.2 (19/44)	NS	NS		NS
CD163+ advanced edges	45.4 (20/44)	NS	NS		NS
TILs (*n*=24)	37.5 (9)	NS	NS		NS
*Mutations*					
At least one	62.9 (44)	*P*=0.06	NS		*P*=0.059
*BRAF*		NS			
+	40 (28)	25			
–	60 (42)	30.9			
*NRAS*		*P*=0.019	NS		NS
+	22.7 (15/66)	53.3	80	66	15.1
–	77.3 (51/66)	19.6	68.2	53.4	3.9
*cKIT* (n=54)		NS			
+	1.9 (1/54)	100			
–	98.1 (53/54)	26.4			

*ORR* objective response rate, *OS* overall survival, *PFS* progression-free survival, *NS* non-significant: ≥0.05, *PD-1* programmed death-1, *PD-L1* programmed death-ligand-1, *TILs* tumour-infiltrating T lymphocytes.

^a^Values are expressed as *n* (%) or median (range)

At least one *BRAF*, neuroblastoma-*RAS* oncogene (*NRAS*) or *cKIT* mutation was found in 62.9% of the lesions and 22.7% were *NRAS*-mutated. Although *NRAS*-mutated lesions (regardless of mutation type) were associated with better ORR than those *NRAS*–, their corresponding OS (66% vs 53.4% at 12 months, *P* = 0.32) and PFS (median of 15.1 vs 3.9 months; *P* = 0.2) were comparable. Non-significant trends towards better ORR and PFS were observed for patients with tumours harboring at least one mutation (Kaplan–Meier plots available in supplementary figures 4 and 8).

Labeling with the anti-PD-L1 antibodies used appeared quite similar. PD-L1-labeled areas were similar for primary melanomas and their metastases (Fig. 1). PD-L1 expression on primary melanomas, but not metastases, was associated with ORR (35.7% for PD-L1+ vs 5% for PD-L1–; *P* = 0.02). Neither PD-L1 status at advancing edges nor the percentage of PD-L1+ tumour cells was associated with ORR, OS or PFS (Kaplan–Meier plots available in supplementary figures 1, 2 and 5). Agreement between primary melanoma and metastases was poor for total tumour-area PD-L1 expression, TIL assessment and CD163+ histiocytes at advancing tumour edge. None of those variables was associated with improved ORR or survival.

CD8+ TILs, seen in 75% of primary melanomas and 57.9% of metastases, were not associated with ORR or OS, regardless of the distribution pattern analysed. More than 10% of CD163+ histiocytes were observed at advancing tumour edges in 68.1% of primary melanomas and 53.8% of metastases. CD163+ expression in primary lesions was associated with better ORR and longer PFS (Kaplan–Meier plots available in supplementary figure 6). Very heterogenous TIL- and PD-L1+-cell distributions prevented subgroup assessment according to Teng’s four-tier–grading.^18^

Interobserver reproducibility was high for PD-L1+ areas in primary melanomas and metastases (ICC = 0.86 and 0.9, respectively), and CD163+-histiocyte analysis at the advancing edges (ICC = 0.78)

**Fig. 2:**
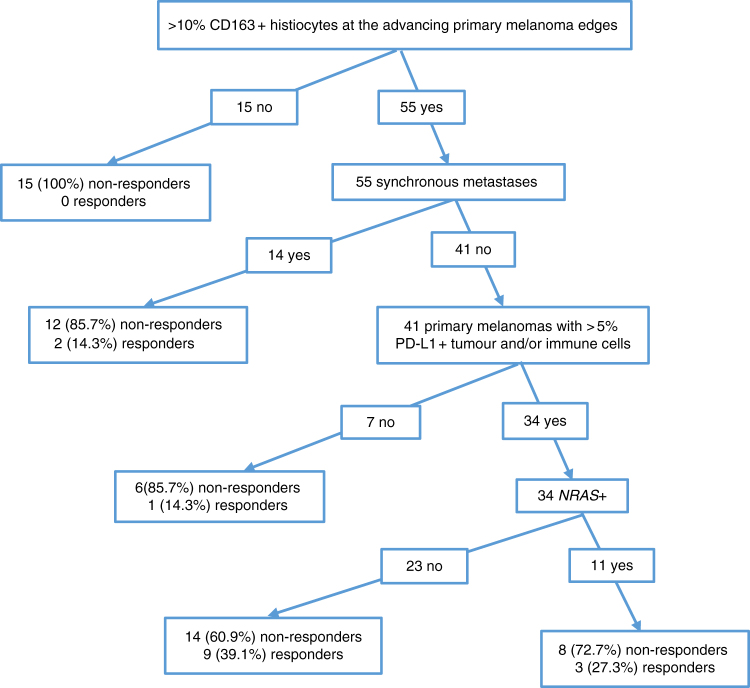
Decision-tree algorithm created via recursive partitioning. This decision tree indicates how well different variables predict 6-month objective response class. Clinicians could easily use this kind of tree for routine care. *PD-L1* programmed death-lig and-1

Significant criteria (*P* < 0.05) were combined to create a decision-tree algorithm (Fig. 2), with 40% sensitivity, 94% specificity, 72.73% PPV and 79.66% NPV.

## Discussion

According to our results, PD-L1 status alone cannot be used as a reliable biomarker of therapeutic response to anti-PD-1 immunotherapy but could be combined with other criteria, e.g., synchronous metastases, > 10% CD163+ histocytes at advancing tumour edges or *NRAS* status (as previously reported^19^).

We observed that PD-L1 was often expressed on histiocytes closely intermingled with tumour melanocytes, which made the distinction between PDL1+ histiocytes and PD-L1+ tumour cells more difficult. The pathophysiological significance of such histiocyte PD-L1 expression remains unclear but has been reported for several cancers.^12^ Evaluating PD-L1-positivity on tumour and immune cells is more reliable and reproducible than tumour cells alone. Although double-labeling was not done, analysing only tumour-cell PD-L1–positivity (with a 5% cut-off) is perhaps less reliable, especially at the advancing tumour edges, where inflammatory immune cells are usually extensively intermingled.^16^

PD-L1 status appears to be relevant in primary melanomas. If that observation is confirmed, it could be helpful, especially because metastases are often difficult-to-access or analyse (e.g. obstacles to evaluating lymph-node metastases include intrinsic PD-L1 expression, particularly sinusal histiocytes).

To the best of our knowledge, CD163+ histiocytic infiltrates at advancing tumour edges have not yet been thoroughly evaluated in melanomas and might be a potentially helpful biomarker. CD163+ histiocytes are usually considered M2 macrophages and associated with poorer outcomes.^14,20^ Although surprising, our results are consistent with histiocytes playing a critical role in immune resistance.

In conclusion, identification of patient subgroups responding less well to anti-PD-1 immunotherapy with our algorithm will not avoid these agents, but might obtain better monitoring, and hence more quickly identify progressive disease. Our findings highlighted the potential biomarker role of combining CD163+ histiocytes in melanomas and their metastases with other variables but need to be validated with a larger prospective cohort, including assessment with available anti-PD-L1 monoclonal antibodies, before definitive conclusions can be drawn.

## Electronic supplementary material

Supplementary figures

## Availability of data and materials

All data supporting the results reported in the article can be found in departments of Pathology of Bordeaux hospital (CHU) and Toulouse Oncopole.
