# Supplementary material for: Clinical, histological and molecular predictors of metastatic melanoma responses to anti-PD-1 immunotherapy
Source: Br J Cancer. 2018 Jul 5;119(2):193–9. doi: 10.1038/s41416-018-0168-9 (PMC6048096; doi:10.1038/s41416-018-0168-9)
Supplement: Supplementary file 1 — Supplementary figures [file 41416_2018_168_MOESM1_ESM.docx]

**SUPPLEMENTARY FIGURES**

**Supplementary figure 1: Kaplan–Meier plots showing the overall survival rates depending on PD-L1 tumor area (cut-off 5%), in primary tumor.**

|  | PD-L1 tumor area <5% |
| --- | --- |
|  | PD-L1 tumor area >5% |

**Supplementary figure 2: Kaplan–Meier plots showing the overall survival rates depending on CD163+ margin histiocytes (cut-off 10%), in primary tumor.**

|  | CD163+ margin histiocytes >10% |
| --- | --- |
|  | CD163+ margin histiocytes >10% |

**Supplementary figure 3: Kaplan–Meier plots showing the overall survival rates depending on metastasis chronology.**

|  | Metachronous metastases |
| --- | --- |
|  | Synchronous metastases |

**Supplementary figure 4: Kaplan–Meier plots showing the overall survival rates depending on NRAS mutational status.**

|  | *NRAS*+ |
| --- | --- |
|  | *NRAS*– |

**Supplementary figure 5: Kaplan–Meier plots showing the progression free survival rates depending on PD-L1 tumor area (cut-off 5%), in metastasis.**

|  | PD-L1 tumor area >5% |
| --- | --- |
|  | PD-L1 tumor area <5% |

**Supplementary figure 6: Kaplan–Meier plots showing the progression free survival rates depending on CD163+ margin histiocytes (cut-off 10%), in primary tumor.**

|  | CD163+ margin histiocytes >10% |
| --- | --- |
|  | CD163+ margin histiocytes <10% |

**Supplementary figure 7: Kaplan–Meier plots showing the progression free survival rates depending on metastasis chronology.**

|  | Metachronous metastases |
| --- | --- |
|  | Synchronous metastases |

**Supplementary figure 8: Kaplan–Meier plots showing the progression free survival rates depending on NRAS mutational status.**

|  | *NRAS+* |
| --- | --- |
|  | *NRAS-* |

**Supplementary figure 9: confusion matrix of the decision tree (figure 2).**

| **Tree answer** | **Objective response** | |
| --- | --- | --- |
|  | **No** | **Yes** |
| **No** | 47 | 12 |
| **Yes** | 3 | 8 |
